# American Medical Association

**Published:** 1869-06

**Authors:** 


### Miscellaneous.

From the Louisville Journal.

### American Medical Association.

THIRD DAY.—Dr. Baldwin, President, in the chair. Reading of the minutes was dispensed with.

On Dr. Parvin's report of Committee on President's Address, your committee desire to present the following report:

We cannot refrain, before entering upon the consideration of the plan recommended by the President for the improvement of medical education, gladly expressing our high appreciation of the general tone of this address, of the broad and catholic spirit which pervades it, finding expression in earnest and eloquent words—in brief, we believe the address worthy the perusal of every member of the profession, in that it was worthy the memorably occasion, and is worthy the annals of medicine.

On the other hand, we cannot refrain, with sadness be it said, from acknowledging the truth of the terrible allegations made against the present condition of medical education, and the little success attending the efforts for improvements in such connection, made during a score of years.

The special recommendation made by the President is in these words:

"I would advise that we appoint a committee of our wisest and best men to digest a plan for one or more National Medical Schools, and to memorialize Congress in behalf of the enterprise. Let the plan embrace as a basis the features presented by the Cincinnati Convention of Teachers; let these schools or universities confer such distinctions and privileges as will be proportionate to the superiority they demand, and such as will make the attainment of their diploma an object of the ambition of those who engage in the study of medicine; let the choice be open to all aspirants, and the appointment or election of professors so guarded as to secure the very highest talent, the most profound learning, with the most fully demonstrated capacity for teaching. Make the salaries of the professors large, and not to depend upon the number of students; and let the Federal Government assume a proper share of the expenses incurred."

Your committee express their hearty approval of this general plan, but suggest that the effort at first should be for the establishment of but a single school, as more feasible, and beside, one such institution would be a model which other medical colleges might in time be induced to imitate in extent, duration and thoroughness of teaching, in rigidness of requirements for the degree of M. D.

We likewise desire to say that when the details of this general plan are thrown into form, there should be the amplest security against the places and the power of such a medical college as designed, ever falling into the hands of politicians or the proteges of politicians. Medicine is higher than politics; broader than political creeds and party platforms.

In conclusion, your committee reiterate the recommendation of the President as to the appointment of a committee for the special purposes referred to.

The President appointed this committee as follows: Dr. N. S. Davis, of Chicago; Dr. F. Gurney Smith, of Philadelphia; Dr. D. H. Storer, of Boston; Dr. E. S. Gaillard, of Louisville; Dr. Joseph Jones, of New Orleans.

On motion, Dr. W. O. Baldwin, of Montgomery, was added to the committee.

The President appointed as delegates to the British Medical Association: Dr. N. Pinckney, U. S. N.; R. R. McIlvain, Ohio; J. F. Hibberd, Indiana; B. Lindsey, D. C.; G. C. Blackman, Ohio. To the Canadian Association: Dr. Alden March, Albany, N. Y.

Dr. Davis read report of meeting of editors and presented the following from the Association of American Medical Editors:

*To the American Medical Association:*—I have been instructed to announce to your honorable body, that those members of your Association in attendance on this annual meeting, after proper consultation, have effected a permanent organization with the title of "The Association of American Medical Editors." The objects of this organization are the cultivation of friendly relations; mutual assistance; community of effort and views, where possible, in a system of receiving foreign exchanges, and sending our own journals abroad; concert of action in support of improvement in the present system of medical education, and of a higher standard of preliminary attainments for those who propose to enter upon the study of medicine; in proposing laws for the proper registration of births, marriages and deaths; in collecting the names of all the regular practitioners in the several States; and in promoting generally the value and efficiency of our periodical medical literature. The Association thus formed is to hold its annual sessions on the day preceding the annual meetings of this body, and in the same localities. Dr. Mitchell of New Orleans, is the Permanent Secretary, and Dr. J. B. Lindsley of Nashville, Tenn., the Assistant Secretary. Congratulating your honorable body on the establishment of another organized power within the ranks of your noble profession,

I remain yours, most truly,

N. S. Davis, Editor,

President of Association of American Medical Editors.

Referred to Committee on Publication.

In the case of a special violation of the code in Louisville, Ky., Dr. Gaillard desired to remove an unfortunate and incorrect impression created in the minds of the members of the Association by one of the delegates from Louisville, (Dr. Yandell,) in regard to a failure on the part of two medical societies in that city to meet promptly and fully an alleged breach of the code of ethics on the part of one of the members of these societies.

Dr. Gaillard stated that he was satisfied Dr. Yandell had no intention of creating a false impression in this connection. The gentleman against whom charges had been preferred was a German of professional proficiency, who recently arrived, and ignorant of the code of ethics, had advertised himself as a specialist in the daily papers, and had sent private handbills to professional men.

As soon as this gentleman was apprised of his fault he had promptly withdrawn his advertisement from the daily papers and had ceased sending the handbills mentioned. The medical societies of Louisville decided that though there had been a breach in the letter of the code of ethics, the gentleman arraigned had no intention of doing what was professionally wrong. These societies, therefore, declined to expel the member against whom these charges had been preferred. Dr. Gaillard thought it due to those societies and to the gentleman offending that this explanation should go upon the records.

The Committee on Nominations unanimously report as follows: For President, George Mendenhall, Ohio; Vice Presidents, Warren Stone, La.; Lewis A. Sayre, N. Y.; F. Gurney Smith, Penn.; John S. Moore, Mo.; Assistant Secretary, William Lee, D. C.; Treasurer, Caspar Wister, Penn.; Librarian, Robert Reyburn, D. C.

Committee of Arrangements—Thomas Antisell, chairman; Robert Reyburn, C. M. Ford, L. W. Ritchie, W. J. C. Duhamel, D. R. Hayner, C. F. Nally.

Committee of Publication—F. Gurney Smith, Pa., chairman; W. B. Atkinson, Pa.; A. J. Semmes, Ga.; Robert Reyburn, D. C.; Caspar Wister, Pa.; H. F. Askew, Del.; Wm. Maybury, Pa.

Committee on Medical Literature—J. J. Woodward, U. S. A., chairman; W. H. Anderson, Ala.; Theophilus Parvin, Ind.; H. A. Johnson, Ill.; C. W. Parsons, R. I.

Committee on Prize Essays—Grafton Tyler, D. C., chairman; N. R. Lincoln, D. C.; N. R. Smith, Md.; G. W. Miltenberger, Md.; W. R. Dunbar, Md.

Committee on Epidemics add the following to fill vacancies—J. K. Bartlett, Wis.; J. B. Jackson, Ky.

Committee on Education—T. G. Richardson, La., chairman; E. W. Jenks, Mich.; E. S. Gaillard, Ky.; W. M. McPheeters, Mo.

Time for meeting, in Washington, first Tuesday in May, 1870.

J. J. Woodward, U. S. A., Chairman.

The report was unanimously adopted:

Dr. Davis offered the following:

*Resolved*, That a special committee of three be appointed by the President to present copies of the resolutions adopted before the several State Medical Societies at as early a period as possible. Adopted.

Dr. Chaille of Louisiana, chairman of the committee, presented a report on Medical Nomenclature, which was received and adopted, and referred to Committee on Publication.

The Committee on the Nomenclature of Diseases have the honor to report that it has examined the "Provisional Nomenclature of the Royal College of Physicians" of London, and is of the opinion that it is desirable for this Association to recommend and adopt the same for general use in this country, with such modifications as, on deliberate consideration, may appear to be necessary. The following resolutions are therefore submitted:

1. *Resolved*, That a special committee of fifteen be appointed by the President to take this subject into deliberate consideration, and to report at the next annual session what alteration, if any, are necessary to adapt the proposed nomenclature to general use in the United States.

2. That this committee be authorized to fill up any vacancies which may occur upon it.

3. That the Committee on Publication be authorized to publish for general distribution one thousand copies of the English and Latin portions of this nomenclature, under the designation of the Proposed Nomenclature, prefacing the same with such remarks as may be deemed necessary to secure the criticism and coöperation of as large a number of American medical men as practicable.

4. That the committee hereby appointed be directed to draw the attention of the Surgeon-General of the Army, of the Chief of the Bureau of Medicine and Surgery of the Navy, and of the Superintendent of the Census, to the question of their official adoption of the proposed Nomenclature; to invite them to appoint whom they see fit to represent them on this committee; and to solicit such coöperation as may be necessary to accomplish the purpose desired, viz.: the final adoption of such nomenclature and classification as will receive the conjoint approval of the official medical bureau of the government and of the general profession.

STANFORD E. CHAILLE, M. D., Chairman.

Committee—S. E. Chaille, La.; J. J. Woodward, U. S. A.; A. B. Palmer, Mich.; F. F. Smith, Penn.; J. F. Heustis, Ala.

The following committee of fifteen was appointed: Francis G. Smith, chairman; J. J. Woodward, U. S. A.; R. F. Michel, Ala.; A. B. Palmer, Mich.; S. E. Chaille, La.; L. P. Yandell, Jr., Ky.; Austin Flint, N. Y.; Alonzo Clark, N. Y.; Geo. B. Wood, Penn.; S. H. Dickson, Penn.; E. Jarvis, Mass.; Theophilus Parvin, Ind.; W. M. McPheeters, Mo.; E. M. Snow, R. I.; N. Pinkney, U. S. N.

The Committee on Medical Education having referred matters at issue to State Medical Societies, Dr. E. S. Gaillard of Louisville, offered the following motion:

*Moved*, That the adoption of a uniform rate of collegiate's fees, $120, being the *minimum*, be accepted as the sentiment and desire of this Association.

Dr. Gaillard stated that he would not trespass upon the time of the Association in speaking upon this motion; that all of the members present were fully informed upon this subject. He said the profession desired to learn the wish and decision of the Association upon this all important question, and he asked a full expression of opinion and a full vote in regard to it.

Dr. Sayre of New York, opposed the resolution, but, on understanding that it did not prohibit an increase of fees, withdrew his objections. He spoke against cheap medical colleges, which allured young men to an imperfect medical education, who were afterward turned back to the plow.

An amendment was proposed by Dr. Logan of Louisiana, to make the minimum $140 instead of $120.

Dr. Mussey of Ohio, opposed the amendment, and stated that the fees of Ohio had to be kept down to accord with the fees of Michigan colleges. The location of the college and the cost of living made the difference. A hundred and forty dollar college is considered good, simply because a hundred and forty dollars is the fee, while other colleges were equally as good where the fees were only eighty dollars. It is impracticable to accomplish this change at once. A new college starts and comes up to the full standard of the old college in the vicinity, and the latter comes down with its fees. The new college must come down, also, in order to maintain itself.

Dr. McPheeters of Missouri, did not agree with Dr. Mussey that it was impracticable to fix the collegiate fees at a minimum of one hundred and twenty dollars. He favored the original resolution, without amendment.

Dr. Palmer of Michigan, alluded to the remarks of Dr. Sayre of New York, disparaging one-horse and cheap colleges. The University of Michigan was established and allowed a donation from the General Government of two townships of land, and it has husbanded its resources and can maintain itself with moderate fees. Under the organic law of the State, citizens of Michigan were entitled to the benefits of the university free of charge, and, as a liberal donation had been made by the General Government, students had been admitted from other States on the same terms. Lately, however, a small fee had been charged for students from other States who received the benefits of the lectures. We come up in fees just as far as we think is for the advantage of the institution, and we do not go beyond that point, because it will diminish the numbers. We are willing to put up the fees for students from other States to one hundred and forty dollars if neighboring States will make the same requirements from their students as we do.

Dr. Palmer then commenced describing the *great advantages of this school*, when Dr. Gaillard called him to order; stating that we were present to discuss principles involved and not to listen to eulogies upon special schools.

Dr. Davis of Illinois: I do not object to discuss the fees, but I do claim that it is out of place to advertise the superior claims of State colleges here. We have had no more illiterate students in our Illinois college than have come to us after one course in the University of Michigan.

Dr. Parvin of Indiana: I move to amend by striking out $140 and inserting $100. If we make the fees of colleges uniform, the next step will be to make the fees of practitioners uniform—the same in villages of the West as in the city of New York—and that is not equitable or practicable.

Dr. Logan of Louisiana, advocated the sum of $140 as the minimum of collegiate fees, and advocated the adoption of his amendment. Lost.

The amendment of Dr. Parvin, to fix the minimum at $100, was also lost.

The resolution was then, as originally presented, unanimously adopted.

Special committee on the relative advantages of Syme's and Pirogoff's mode of amputating at the ankle—Dr. G. A. Otis, U. S. A., chairman; Dr. J. M. Holloway, of Louisville, Ky.

Proposed by J. J. Woodward. Approved.

Dr. Bemis presented from Dr. John Waters of St. Louis, Mo., a paper on the Doctrines of Force—Physical and Vital.

Dr. Toner, D. C., moved that a Committee on Variola be appointed—Dr. J. Jones, chairman. Adopted.

Dr. Pinckney, U. S. N., made statements concerning relative grades of rank. The paper was ordered to be spread upon the minutes.

Association adjourned to meet at 9 o'clock A. M., May 7th.

FOURTH DAY.—The Association met at 9 o'clock, Dr. Baldwin in the chair.

Reading of the minutes omitted.

Dr. Joseph Jones of Louisiana, presented a number of specimens of pathology, anatomy and natural history. The explanations were very interesting, and received with applause.

On motion of Dr. Garrish of Kentucky, the thanks of the Association were tendered to Dr. Jones.

On motion of Dr. F. G. Smith of Pennsylvania, the following resolution was unanimously adopted by a vote of the members present, standing as a mark of respect:

*Resolved*, That the thanks of this Association are justly due and are hereby tendered to the President for the uniform kindness and courtesy with which he has presided over its deliberations, and to the Committee of Arrangements, the physicians, and citizens of New Orleans for the generous hospitality and fraternal kindness with which we have been received and treated during our sojourn in their city, with the assurance that the memories of this visit will always be among the brightest and most enduring of our lives.

*Resolved*, That we also present our thanks to the various railroad and steamboat companies who have so liberally extended to us facilities of transportation, and to the daily press for their efficient aid in reporting the proceedings of this meeting.

On motion of J. P. Moore of Mississippi, the following preamble and resolution was adopted:

*Whereas* the contract system is contrary to medical ethics,

*Resolved*, That all contract physicians, as well as those guilty of bidding for practice at less rates than those established by a majority of regular graduates of the same locality, be classed as irregular practitioners.

The following reports of the sections followed:

Section on Meteorology, Medical Topography and Epidemics reported. Papers accepted and referred to the Committee on Publications.

Sections on Practical Medicine and Obstetrics reported and were accepted, and referred to Committee on Publication.

And the report on Training of Nurses was accepted and the resolutions adopted.

Section on Medical Jurisprudence, Hygiene and Physiology reported. Report accepted and referred to Committee on Publication.

Section on Surgery proposed that their report be received without formality and be referred to the Committee on Publication. Adopted.

After being read, the report was accepted and ordered to be published.

Section on Psychology, the same disposition.

The President appointed Dr. J. M. Toner a committee of one, at Washington, D. C., to assist the Librarian of Congress to keep the books of the Association.

On motion for adjournment, the President delivered this address, which was unanimously accepted and ordered to be published in the transactions of the Association.

Gentlemen:—Before I submit the motion just made, and which, when adopted, will practically close my official relations to this body, allow me to return you my most cordial and grateful thanks for the universal kindness which I have received at your hands. Whatever my future lot in life may be, the world holds no honors which to me can equal those conferred by you. The fraternal good will which has so conspicuously marked your deliberations has been to me a matter of infinite satisfaction and pride, and will not be the least among the grateful memories which will gladden my heart as I may hereafter review the incidents of my official connection with you.

To win your judgment and approval, to hold up the dignity of fellowship, the usefulness of association and the interest and prosperity of the profession at large have certainly occupied my most anxious thoughts since my elevation to this position; yet to cherish and promote the intimate and cordial relations of friendship between the individual members of this Association against all sectional distinctions or geographical lines, has also been among the chief objects of my ambition and the earnest desires of my heart. Could I now believe that my efforts have contributed in the slightest degree to enlarging that harmony of sentiment and fraternal feeling which has been so apparent throughout this meeting, I

should feel that I had commenced at least to make some return for the great honor and kindness received at your hands.

It now only remains for me, gentlemen, to again express to you my thanks, to wish you a safe return to your homes and labors, a happy reunion with your friends and families, and to pronounce that sad word, over which the heart of friendship would fain linger, as I bid you an affectionate farewell.

W. O. BALDWIN, President A. M. A.

The Convention adjourned to meet in Washington, D. C., the second Tuesday in May, 1870.

